# Practical Notes on Diagnosis and Treatment

**Published:** 1910-03-12

**Authors:** 


### Practical Notes on Diagnosis and Treatment.

### Insomnia due to Drugs.

It is sometimes overlooked in considering the insomnia of invalids that this may be due to drugs given for other purposes, such as caffeine theobromine or strychnine.

### Bier's Passive Congestion.

One of the great failures of passive congestion is in erysipelas, in which condition it does no good at all. The inflammation will travel as high as, or will even pass, the bandage which has been used to produce the passive congestion.—*Mr. E. M. Corner.*

### Baldness.

There is a strong case to be made out for the hard hat being the cause of baldness by compressing the nutrient arteries of the scalp. It would appear that the associated phenomena, such as seborrhœa, thinning of the scalp, etc., are all due to local anæmia, and that the anæmia and debility, when present, are considered by supporters of the hat theory to be merely the associated effects of town life.

### Alcohol.

Under different circumstances alcohol acts in contrary directions; it may be said to prove beneficial when its effect is to bring the heart's action towards the normal. In febrile cases it brings about a reduction of the number of beats, while in heart failure, with feebleness and infrequency, alcohol tends to increase the rapidity and force of the heart's action. The continued exhibition of alcohol in chronic diseases of the heart is to be discouraged, and in acute forms of illness alcohol should not be given before a real necessity for its use arises.—*Dr. Essex Wynter.*

### Creosote.

Prolonged use of creosote has led me to the conclusion that it does exert a beneficial—even curative—influence in the more chronic forms of pulmonary tuberculosis. More particularly, cough and expectoration are often reduced remarkably, secretion being lessened and thereby the need for cough removed. The gastro-intestinal tract also benefits, while appetite improves and complicating diarrhœa is frequently relieved. Disturbing effects have been observed, but have not occurred in my experience when pure beechwood creosote has been used. Still more rarely does renal trouble ensue, but an eye must be kept on the urine nevertheless.—*Dr. R. W. Philip.*

### Hæmorrhoids.

Plentiful water drinking is indicated in this disorder, and a course of aperient mineral water is the most advantageous.—*Dr. C. D. Phillips.*

### Guaiacum in Gout.

In spite of the high commendation formerly given to the use of guaiacum in chronic articular gout, this drug is but little used; but it undoubtedly does give great relief to the muscular pains of persons subject to gout.

### Water Beds.

As a mild but effective method of applying the cold-water cure to cases of fever, water beds are well adapted. By running cold water through the bed continuously the temperature of the patient lying on it may be to some extent controlled.—*Dr. C. D. Phillips.*

### Hysterical Paralysis.

An unqualified diagnosis of hysterical paralysis in children should rarely be made. Even when the manifestations of hysteria are obvious, almost invariably some antecedent morbid condition, which may still remain uncured, has set them up. Hysterical symptoms may be caused by incipient organic disease, for example, hip joint disease.—*Dr. L. Guthrie.*

### Iodine and Iodides.

Owing to the expense of potassium iodide it has been suggested that small doses of tincture of iodine, well diluted, would have the same therapeutic effect. Iodine appears to be absorbed in the stomach, partly as an albuminate, but chiefly as sodium iodide, and this combination with sodium occurs also to some extent in the blood. Alkaline iodides are either absorbed unchanged or as iodide of sodium.

### Spinal Douching.

The water used should, to begin with, have a temperature not below 80° F., and be gradually cooled down. If commenced too cold it may give rise to headache or giddiness. The spinal cord appears to be directly stimulated by the shock of the cold water, and the stimulus is reflected to the peripheral and visceral nerves, notably the sympathetic ganglia. This bath is useful in functional torpor, with numbness or slight paralysis of limbs, constipation and phosphaturia, producing a bracing effect and a pleasant glow.

